# Fluoride Consumption and Its Impact on Oral Health

**DOI:** 10.3390/ijerph8010148

**Published:** 2011-01-19

**Authors:** María Dolores Jiménez-Farfán, Juan Carlos Hernández-Guerrero, Lilia Adriana Juárez-López, Luis Fernando Jacinto-Alemán, Javier de la Fuente-Hernández

**Affiliations:** 1 Laboratorio de Inmunología, División de Estudios de Posgrado e Investigación, Facultad de Odontología, Universidad Nacional Autónoma de México, Mexico City, DF, C.P. 04510, Mexico; E-Mails: mjifarfan@yahoo.com.mx (M.D.J.-F.); aeroluis81@hotmail.com (L.F.J.-A.); 2 Facultad de Estudios Superiores-Zaragoza, Universidad Nacional Autónoma de México, Mexico City, DF, C.P. 09230, Mexico; E-Mail: liadju@yahoo.com (L.A.J.-L.); 3 Departamento de Salud Pública Bucal, División de Estudios Profesionales, Facultad de Odontología, Universidad Nacional Autónoma de México, Mexico City, DF, C.P. 04510, Mexico; E-Mail: fuente@servidor.unam.mx (J.F.-H.)

**Keywords:** dental fluorosis, dental caries, fluoride urinary excretion

## Abstract

**Objective:**

The purpose of this study was to evaluate caries and dental fluorosis among Mexican preschoolers and school-aged children in a non-endemic zone for fluorosis and to measure its biological indicators.

**Methods:**

DMFT, DMFS, dmft, dmfs, and CDI indexes were applied. Fluoride urinary excretion and fluoride concentrations in home water, table salt, bottled water, bottled drinks, and toothpaste were determined.

**Results:**

Schoolchildren presented fluorosis (CDI = 0.96) and dental caries (DMFT = 2.64 and DMFS = 3.97). Preschoolers presented dmft = 4.85 and dmfs = 8.80. DMFT and DMFS were lower in children with mild to moderate dental fluorosis (DF). Variable fluoride concentrations were found in the analyzed products (home water = 0.18–0.44 ppm F, table salt = 0–485 ppm F, bottled water = 0.18–0.47 ppm F, juices = 0.08–1.42 ppm F, nectars = 0.07–1.30 ppm F, bottled drinks = 0.10–1.70 ppm F, toothpaste = 0–2,053 ppm F). Mean daily fluoride excretion was 422 ± 176 μg/24 h for schoolchildren and 367 ± 150 μg/24 h for preschoolers.

**Conclusions:**

Data from our study show that, despite values of excretion within an optimal fluoride intake range, the prevalence of caries was significant in both groups, and 60% of the 11- to 12-year-old children presented with dental fluorosis. In addition, variable fluoride concentrations in products frequently consumed by children were found.

## 1. Introduction

The halogen fluoride is well distributed throughout the Earth and never occurs in a free state in Nature. Fluorine exists only in combination with other elements as fluoride compounds, which are constituents of minerals in rocks and soil [1]. Fluoride accumulates in the hard tissues of the body and is known to play an important role in the mineralization of bones and teeth.

Dental caries are considered a significant dental public health problem in Mexico and are often observed in low-socioeconomic status populations [2]. While fluoride is accepted as an effective method to prevent caries, the excessive consumption of fluoride can put bones and teeth at risk of developing fluorosis [3]. Fluoride ingested during dental development, until the age of six years, may promote the development of fluorosis. Fluorosis is viewed as primarily affecting permanent dentition, and very high fluoride levels (>10 ppm) are required in drinking water for the fluoride to cross the placental barrier and affect primary dentition. Multiple mechanisms, including direct fluoride-related effects on ameloblasts (secretory and maturation phases), indirect fluoride-related effects on the forming matrix (nucleation and crystal growth in all stages of enamel formation), and calcium homeostasis, can result in dental fluorosis depending on the dose and duration of fluoride exposure [4,5].

In clinical terms, dental fluorosis may result in varying degrees of structural damage, superficial porosity, and loss of continuity of the dental enamel layer [6]. Dental fluorosis may be more than a cosmetic defect if enough fluorotic enamel is fractured and lost, causing pain, adversely affecting food choices, compromising chewing efficiency, and requiring complex dental treatment. Chronic poisoning by fluoride consumption is a global public health issue that is principally observed in areas with above-optimal fluoride levels in the drinking water [7,8]. In Mexico, the fluoride levels in ground water vary substantially among the different regions [8–11]. Due to concern with the increase in the prevalence of caries and fluorosis in Mexico, several studies have been performed to identify risk factors for these conditions. These studies have employed different methodologies and have followed different populations with variable sources of fluoride exposure. Specifically, dental fluorosis in nonendemic areas has been associated with the consumption of fluoridated drinks and foods and the misuse of toothpastes, mouth rinses, gels, drops, and tablets [12]. The Mexican Ministry of Health implemented the National Program for Salt Fluoridation to prevent caries, but the regulation and distribution of fluoridated salt in Mexican areas with endemic and non-endemic dental fluorosis is paradoxical and controversial [13]. Although black beans, yellow tortillas and nopales are part of the basic diet for Mexicans, some habits have changed because of the introduction of many national and imported brands of foods and drinks.

It has been reported that some endemic Mexican areas for dental fluorosis have a dental caries prevalence of 48.6 percent among children 12 to 15 years of age (DMFT = 1.15). In terms of severity, 9.6 percent of those adolescents had DMFT ≥ 4, and 1.7 percent had DMFT ≥ 7. Meanwhile, 81.6 percent had very mild to severe dental fluorosis (CDI = 1.78) [14,15]. On the other hand, one study conducted in an area considered to be non-endemic for dental fluorosis observed prevalences of dental caries ranging from 71.4 percent to 90.5 percent among 12-year-old children (DMFT = 2.78 to 4.64) [16]. Another study found DMFT = 3.24 and a caries prevalence of 82 percent (DMFT > 3 was 47.8 percent, and DMFT > 6 was 9 percent) in schoolchildren [17]. Meanwhile, fluorosis prevalence was 56.3 percent (CDI = 0.7) among children 6 to 9 years old [14], and 89.5 percent (CDI = 2.67) among children 12 to 15 years old [18].

Thus, the purpose of this study was to evaluate caries and dental fluorosis among Mexican preschoolers and schoolchildren in a non-endemic zone for fluorosis and to measure its biological indicators.

## 2. Experimental Section

Ten elementary and five kindergarten schools were randomly selected from the eastern area of Mexico City. First, all students (2,122) from preschool to sixth grade were considered. After applying the inclusion criteria, which specified that all children be clinically healthy, had lived in the area since birth, and had signed the informed consent form, the final sample comprised 1,942 children: 1,569 11–12-year-olds and 373 4–5-year-olds. The exclusion criteria included medical treatment during the study and use of dental appliances. For the determination of urinary fluoride levels, only 205 randomly selected children were included (10 percent of the total sample).

### 2.1. Oral Examination

The World Health Organization’s (WHO) criteria for fluorosis and dental indexes [19], Dean’s Community Index (CDI), DMFT, DMFS, dmft, and dmfs (*D* and *d*, decayed; *M* and *m*, missing; *F* and *f*, filled; *T* and *t*, teeth; *S* and *s*, surfaces), were used by two experienced examiners with at least five years of postgraduate clinical training at the start of the study. After a directed brushing, an intra-oral examination was performed in daylight, using portable dental chairs, plane mirrors and dental probes with rounded tips. The subjects’ teeth were dried with sterile cotton for better visibility before the examination. Cohen’s Kappa values for diagnosing dental caries were 0.86 and 0.92 for intraexaminer agreement and 0.90 for interexaminer agreement. Cohen’s Kappa values for diagnosis of dental fluorosis were 0.76 and 0.78 for intraexaminer and 0.87 for interexaminer.

### 2.2. Questionnaire on the Use of Fluoride-Containing Products

A questionnaire was delivered to the parents of each child included in the study. The questions were previously evaluated with regard to feasibility and reliability through a pilot study administered to 50 persons. We collected the following data: age at which the children began brushing, how many times they brushed their teeth each day; the type of toothpaste used (fluoridated or non-fluoridated); the amount of toothpaste applied to the toothbrush; the use of products containing fluoride, such as mouth rinses, drops, tablets, gels, or solutions; participation in dental health programs during the first six years; and consumption of fluoridated salt and manufactured beverages (bottled water, juices, nectars and carbonated drinks).

### 2.3. Fluoride Ion-Selective Electrode Method

This technique was used for assessing the fluoride concentration in urine samples, water collected from the children’s home, juices, nectars, carbonated soft drinks, bottled water, toothpaste, and table salt. We used an Orion 720A potentiometer equipped with a combined Termo Orion 9609BN electrode for fluoride. A calibration curve was constructed based on standard solutions from 0.01 to 10 ppm F (TISAB II, 50:50; TISAB III, 90:10). Based on the Nerdst equation, mV data were registered in the calibration slope with a minimal value of r^2^ = 0.999.

### 2.4. Collection and Analysis of the Urine Samples

At the beginning of the study, we requested multiple urine samples from randomly selected children: 155 11- to 12-year-old children and 50 4- to 5-year-olds. The instructions for the correct method of collection were given to the parents. Five previously washed plastic bottles (thrice with deionized water) were delivered to each child. Each bottle was labeled with the name and age of the child. Parents were instructed to register the date and hour of each respective collection and to store the urine at 4 °C until the next day. Samples were sent to the laboratory, and pH and total volume were immediately measured. Samples from children who refused or lost part of the 24-h urine collection were excluded. Additionally, we excluded urine samples with a volume lower than 290 mL or with a flow rate greater than 9 mL/h [20]. Later, triplicate aliquots of 20 mL were prepared and mixed with 2 mL of TISAB III. The fluoride concentration of each sample was determined by the fluoride ion-selective electrode. The volume of each sample was divided by the time between each urine collection; this volume was then multiplied by the concentration of fluoride in the sample. Individual values for each child were added and averaged.

### 2.5. Collection and Analysis of Toothpaste Samples

Toothpaste was purchased from supermarkets and grocery stores in the analyzed area. We recorded brand, presentation (for children or adults), lot number, and expiration date. One gram of toothpaste was mixed with 2.5 mL of deionized water and centrifuged at 5,000 rpm for 30 min. Fluoride concentration was assessed in triplicate using the fluoride ion-selective electrode. Calculations of the percentage of fluoride were made as follows: $\%\text{F}=[(\text{ppm F})\;(\text{weight of the sample}+\text{weight }\text{of the buffer solution}\times 100)]\;(\text{weight of the sample}\times 10^{6})$.

### 2.6. Collection and Analysis of Table Salt

Samples were obtained from supermarkets and grocery stores in the analyzed area. Brand, type of package (cardboard or plastic bag), source (land or marine), place of manufacturing, lot number, and expiration date were recorded. Samples were stored at room temperature (20–25 °C) and analyzed according to NOM-040-SSA-1981 [21]. Briefly, 2 g of table salt were weighed and dissolved in 25 mL of deionized water. Fluoride concentration was determined with the fluoride ion-selective electrode.

### 2.7. Collection and Analysis of Home Water, Juices, Nectars, Bottled Water, and Carbonated Drinks

Three previously washed plastic bottles were provided to the parents to collect three water samples from their homes on three different days. These samples were stored at 4 °C until the chemical analysis. Juices, nectars, bottled water, and carbonated drinks were purchased from supermarkets and groceries stores of the analyzed area. Brand, type of package (plastic, glass, or cardboard), flavor, lot number, and expiration date were recorded. Fluoride concentration was determined in triplicate using the fluoride ion-selective electrode.

### 2.8. Statistical Analysis

Study data were collected using Microsoft Excel and analyzed with SPSS 11.0 (Chicago, Illinois). Frequencies, means, standard deviations, and percentages were obtained. Chi-squared tests were used for comparing groups with and without dental fluorosis. Association among caries indexes, CDI, and total fluoride excretion was assessed by bivariate logistic regression. To analyze the dichotomized variables *fluoridated products* and *attends dental fluorosis*, odds ratios were used. Table 1 shows the percent distribution of dental fluorosis in Mexican children from a non-endemic area. Table 2 establishes the difference between children with no fluorosis and fluorosis, showing minor index of DMFT/DMFS in fluorosis patients. Table 3 shows the speed of fluoride excretion in urine samples determinated at different times of collection in Mexican children from a non-endemic area.

## 3. Results and Discussion

The eastern area of Mexico City (Iztapalapa) is located 2,240 m above sea level and has a predominantly low-income population. This area represents 20 percent of the entire population of Mexico City [22]. We studied 1,569 11- to 12-year-old children and 373 4- to 5-year-old preschoolers from this area.

### 3.1. Prevalence and Severity of Caries and Dental Fluorosis

According to the fluoride concentration in the water, Mexico City is considered a non-endemic zone for dental fluorosis [23]. Although a community salt fluoridation program exists and is considered effective, our results show that only 22 percent of the analyzed preschoolers and 29.5 percent of the older schoolchildren were caries-free. We found that 72.6 percent (n = 1,139) of 11- to 12-year-olds presented with dental caries (DMFT = 2.66 ± 2.40 and DMFS = 3.95 ± 4.18). Seventy-eight percent of the preschoolers (n = 290) presented with dental caries (dmft = 4.85 ± 4.0 and dmfs = 8.80 ± 9.0). Although these values represent a 15- to 25-percent reduction in the incidence of dental caries from previous years [7], we also observed dental fluorosis in 60.1 percent of the 11- to 12-year-olds (CDI = 0.96 ± 0.58) (Table 1). Thus, according to the Dean and Murray criteria (scores above 0.6), this could be considered a public health problem [24]. Additionally, statistical differences were found between DMFT and DMFS indexes among the 11- to 12-year-olds with and without fluorosis (the very mild criterion was the most frequent degree of dental fluorosis registered).

We observed that more that 70 percent of preschoolers and schoolchildren have dental caries, and children with fluorosis presented a lower DMF than those without fluorosis. Thus, the programs aimed at preventing dental caries seem to be effective, but it is necessary to reconsider the role of other variables, such as nutritional status and total ingestion and excretion of fluoride, to develop the optimal dose of fluoride that is suited to patients’ needs. Additionally, other environmental and geographical factors should be evaluated, such as geographical location, weather and altitude [11,25–27].

### 3.2. Urinary Excretion of Fluoride

Approximately 35 to 50 percent of the fluoride absorbed each day by young or middle-aged adults is assimilated by hard tissues within 24 hours, and renal excretion is the predominant route for the removal of inorganic fluoride from the body [28]. Thus, a useful epidemiological tool for monitoring fluoride ingestion is urinary fluoride excretion [29]. We collected 743 urine samples from 155 children 11 to 12 years of age and 198 urine samples from 50 preschoolers 4 to 5 years of age. The mean fluoride excretion for the older children was 0.58 ± 0.29 ppm F (0.14 to 2.36 ppm F), and the total fluoride excretion over 24 h was 422 ± 176 μg/F per day (mean rate of urinary fluoride flow was 25.57 ± 18.89 μg/F/day) (Table 3 shows the urinary fluoride flow at different times of day). The mean F concentration of the 4- to 5-year-old children was 0.84 ± 0.4 ppm F (0.24 to 2.45 ppm F), with a total 24-h fluoride excretion of 367 ± 150 μg/F per day. The mean rate of urinary fluoride flow was 22.30 ± 7.40 μg/F/day. These values are considered by the WHO to be indicative of optimal fluoride usage. Provisional standards for urinary fluoride excretion and concentration are 360–480 μg/F per day for 3- to 5-year-olds and 600 μg/F per day for 10- to 14-year-olds [29]. Multiple logistic regression showed no association among caries indexes, CDI, and total fluoride excretion.

Our results are in agreement with values found in other countries with fluoridated salt programs, such as Switzerland, France, and Jamaica [30–32]. The mean 24-hour urinary fluoride excretion was similar to those found by Rugg-Gunn *et al.* and Warpeha *et al.* [31,33]. Another study from an optimally water-fluoridated area reported values lower than ours [34]. Nevertheless, considering the fractional urine excretion of fluoride (FUFE) reported in children aged three to six years [35], we expect that our preschoolers ingested approximately 1.1 mg F per day or 0.05 mg/kg body weight. This value is similar to those found in a Peruvian study in which the dietary fluoride intake was estimated by the “duplicate plate method” among children receiving fluoridated salt [36]. This calculation is important. Even though our preschoolers had an optimal intake during the dental development stages, we observed that 60 percent of the 11- to 12-year-olds had dental fluorosis and caries. The dose-response association between fluoride intake and dental fluorosis in permanent teeth suggests that the critical limit of 0.05–0.07 mg/kg body weight is not safe for Mexican children and should be revised in further research [37]. Likewise, some medical conditions, including calcium deficiency, acidic-alkaline balance disorders, urinary flow disturbances, and renal management of fluorides and diet over the longer term, could also affect development of dental fluorosis [38,39].

### 3.3. Use of Fluoride-Containing Products and Fluoride Concentration in Toothpaste

Only 68 percent (n = 1,226 parents) of parents responded to our questionnaire, and 96 percent of them reported using fluoride-containing toothpaste. According to the figures revealed in the questionnaire, 65 percent reported using the entire surface of the toothbrush in indicating the amount of toothpaste used; 45 percent reported that their children had started brushing before they were six years of age, and 53 percent brushed their teeth twice per day. When we considered the amount of dentifrice used and the age and frequency of early brushing, a significant association with dental fluorosis was found (OR = 1.91 (0.41–5.9); *X*^2^ = 13.09, p = 0.003). Of the studied children, 58 percent had never participated in a fluoride preventive program based on fluoridated mouth rinses, drops, professional administrations, self-administered fluoride gels, or any other fluoride supplement. Only 6 percent reported that their children used fluoridated solutions in drops, and 49.4 percent had previously received fluoride through topical applications to their teeth. The mean fluoride concentration of the 65 brands of toothpaste analyzed was 751.30 ± 550.80 ppm F (0 to 2,053 ppm F). Infant toothpastes represented 16.9 percent of the total sample, and their mean fluoride concentration was 563.40 ± 349.80 ppm F (0 to 1,153 ppm F). Overall, 44.6 percent of the toothpastes were manufactured in Mexico, with fluoride concentrations ranging from 0 to 2,053 ppm F (879.0 ± 599.20 ppm F). Imported toothpastes presented concentrations of 619.70 ± 461.70 ppm F (0 to 1,610 ppm F). Of the infant toothpaste samples manufactured in Mexico, 45.5 percent were above the Mexican regulation (730 to 1,153 ppm F) [20]; 12.3 percent of the infant toothpastes presented concentrations above 1,500 ppm F. Interestingly, 42.8 percent were manufactured in Mexico and had values from 1,760 to 2,053 ppm F. Approximately 95 percent of children between four and five years of age swallow some amount of toothpaste during brushing because of its pleasant flavor [40]. Ninety-six percent of the children analyzed in our study used fluoridated toothpastes containing 1,000 ppm F or more. The use of some fluoridated supplements during their first years of life was also reported. Approximately 53 percent of the studied children brushed their teeth twice per day, and 65 percent covered the entire toothbrush’s active surface with toothpaste. Because the most critical time to avoid fluoride exposure corresponds to the first six years of a child’s life, dental professionals should provide orientations to parents on the correct use of fluoride-containing products, particularly for those used by children under six years of age.

### 3.4. Fluoride Concentration in Table Salt

We analyzed 44 table salt brands, obtaining a mean of 129.8 ± 144.9 ppm F (0 to 485 ppm F). Fluoride concentration was printed on the package of 39 samples (88 percent). There were 15 products of marine origin (34.1 percent), 11 of land origin (25 percent), and the other 18 (40.9 percent) did not indicate their source. Overall, 9.1 percent of the brands were imported, and in 27.3 percent, fluoride was not detected. The labels of 22 samples (50 percent) indicated that fluoride concentration was in agreement with the Mexican norm (between 200 to 250 ppm F/kg) [21]. Our analysis showed that fluoride concentration ranged from 1 to 124 ppm; eight samples (18.2 percent) contained 125–199 ppm F; three (6.8 percent) contained 200–250 ppm F; and nine samples (20.4 percent) contained 251–485 ppm F. Comparing our results with the Mexican norm, which states that fluoride content for human consumption should be within 200–250 ppm F, we found that only 3 samples (6.8 percent) contained the fluoride concentration established in this regulation, 33 (75 percent) were below the norm, and 8 (18.2 percent) showed fluoride concentrations above the Mexican guidelines (21). Salt products of marine origin had a fluoride concentration of 145.46 ± 168.81 ppm; and the fluoride concentration in the land samples was 136.82 ± 156.43 ppm. Additionally, the fluoride concentration in domestic salt was 142.8 ± 145.74 ppm. None of the four imported salt samples contained fluoride.

Comparing the fluoride concentration printed on the package to that found in this study, the fluoride content was incorrectly labeled in 92 percent of the analyzed samples of table salt. The questionnaire applied in our study disclosed that 74 percent of the parents used fluoridated salt for cooking, but our results indicate that customers are not given reliable information about the quantity of fluoride they are consuming. Fluoride ingestion from foods is a very important factor to take into account when dental fluorosis studies are performed. Mexico was the seventh country to implement a table salt fluoridation program. Previously, Jamaica, France, and Costa Rica reported a reduction in dental caries after its implementation [13]. Data obtained from a Mexican population living in a non-endemic fluorosis zone indicated that since the implementation of the salt fluoridation program, dental caries declined by 43 percent (the mean DMFT index was 4.39 in 1988 and 2.47 in 1997) in 12-year-old children [41]. These figures support the concept that fluoride reduces dental caries in the population. Nevertheless, the results of previous studies show that the consumption of fluoridated water in addition to fluoride-containing products may promote an increased development of dental fluorosis lesions, even in people living in regions considered to be non-endemic areas [42–44].

### 3.5. Fluoride Concentration in Home Water, Bottled Water, Juices, Nectars, and Carbonated Drinks

Some investigations have suggested that the increased consumption of bottled water, soft drinks and juices prepared with fluoridated water may be a significant source of systemic fluoride, and thus these drinks have been implicated as risk factors for dental fluorosis in young children [45,46]. In non-endemic areas of dental fluorosis, it has been observed that exposure to other sources of fluoride aside from drinking water should be considered [47]. We analyzed 155 home water samples, obtaining 0.18 to 0.44 ppm F (0.27 ± 0.06 ppm F). Previously, we reported that the water supply in Mexico City and the Metropolitan area registers values ranging from 0.26 to 1.38 mg/L [43]. In addition, our results on the fluoride level in the water supply were higher than data reported in other non-endemic Mexican populations [25]. This variability could be related to factors such as season and the geographical location of the water supply prior to distribution.

The fluoride content of beverages prepared with water varied among products made by different manufacturers as well as among similar products made by the same company [46]. We analyzed 20 different samples of bottled water, 101 nectars, 105 juices, and 57 bottled carbonated soft drinks (fruit and cola). Bottled waters had 0.08 to 0.37 ppm F (0.21 ± 0.08 ppm F), values that comply with the Mexican regulations [48]. Juices and nectars contained 0.08 to 1.42 ppm F (0.67 ± 0.38) and 0.07 to 1.31 ppm F (0.44 ± 0.35), respectively. Fruit carbonated soft drinks had a fluoride concentration of 0.11 to 1.70 ppm F (0.41 ± 0.34), and cola drinks showed values of 0.10 to 1.62 ppm F (0.49 ± 0.41). Of the juice samples, 15 percent had values over 1 ppm F (grapefruit, pineapple, apple, and prune flavors). Mixed fruits, pear and guava nectar flavors, had values from 1.22 ppm F to 1.42 ppm F. Variability in fluoride concentration among beverages is primarily determined by the fluoride content of the water used during manufacturing and can be affected by the fruit used. We found wide variations in fluoride concentrations in all of the fruit-based beverages tested (0.07 to 1.70 ppm F). In a Mexican zone for endemic dental fluorosis, fluoride levels in bottled water of 0.27 to 1.01 ppm F, in carbonated soft drinks of 0.33 to 3.71 ppm F and in apple juice of 0.2 to 2.9 ppm F have been reported [49]. These differences in fluoride concentrations could be related to the fact that the drinking water in Mexico City has lower fluoride concentrations [43]. The wide ranges of fluoride concentrations in bottled water and other beverages make it difficult to assess the actual fluoride intake by the population. Mexico is considered to rank first place worldwide in the consumption of carbonated soft drinks [50]. Our questionnaire revealed that 94 percent of the children consumed bottled water, nectars, juices or carbonated drinks at least once a week. In infancy, the main fluoride sources are thought to be from commercially available beverages and foods used during weaning, a period coinciding with the stage of mineralization in several developing permanent tooth crowns [51]. Our results demonstrate that bottled drinks have fluoride, and some of them contain more than 1 ppm F, exceeding the standard recommendations [23,52]. It is important that labels should contain all the nutritional information about the product, including fluoride concentration. The need to identify areas with low, recommended, and high levels of fluoride in drinking water or food cannot be underestimated.

## 4. Conclusions

Fluoride continues to be the cornerstone of dental caries prevention throughout the world, and there are a variety of sources of fluoride that may contribute to the dietary intake of fluoride. Even though Mexico City is considered a non-endemic area for dental fluorosis according to its low concentration of fluoride in drinking water, the children in our study presented epidemiological indicators of overexposure to fluoride. Our data revealed a urinary excretion within the normal limits established and reported by other authors, but epidemiological indexes showed simultaneously high prevalences of caries and dental fluorosis. Because our knowledge is incomplete regarding the amount, duration, and timing of fluoride ingestion that can result in dental fluorosis, however, further research is clearly needed before definitive recommendations can be made regarding the use of fluorides, including the recommended dietary intake of fluoride. Further longitudinal studies are needed to determine the safe fluorine dose for Mexican children, taking in account age, nutritional status, altitude, geographical location and weather, among other factors.

## Figures and Tables

**Table 1:** Distribution of dental fluorosis in 11- to 12-year-old.

CDI*	n	percent
0- No fluorosis	70	4.40
1- Doubtful	555	35.40
2- Very mild	662	42.20
3- Mild	270	17.20
4- Moderate	11	0.70
5- Severe	1	0.06

* CDI: Dean’s Community Index.

**Table 2:** DMFT and DMFS in 11- to 12-year-old Mexican children from a non-endemic area for dental fluorosis.

	N	DMFT	Var	DMFS	Var
**No fluorosis**	625	4.06 ± 4.05	16.43	2.80 ± 2.50	6.27
Fluorosis (very mild to severe)*	944	3.48 ± 3.72°	13.85	2.54 ± 2.39Ŧ	5.71

* Except doubtful degree;

° *X*^2^, p = 0.036;

Ŧ *X*^2^, p = 0.41.

**Table 3:** Speed of fluoride excretion in urinary samples.

Time	Volume collected (mL)	Fluoride concentration (mg/L)	Speed of excretion (μg/L)	pH
Morning (7:00–14:00 h)	237.93	0.54	21.19	6.1
Afternoon (14:01–20:00 h)	154.92	0.58	23.14	6.2
Evening (20:00–6:59 h)	170.97	0.60	34.22	6.3
